# An iRGD‐conjugated photothermal therapy‐responsive gold nanoparticle system carrying siCDK7 induces necroptosis and immunotherapeutic responses in lung adenocarcinoma

**DOI:** 10.1002/btm2.10430

**Published:** 2022-10-27

**Authors:** Rui Cai, Meng Wang, Meiyuan Liu, Xiongjie Zhu, Longbao Feng, Zhongjian Yu, Xia Yang, Zhiwu Zhang, Huili Guo, Rui Guo, Yanfang Zheng

**Affiliations:** ^1^ Department of Medical Oncology Affiliated Cancer Hospital and Institute of Guangzhou Medical University Guangzhou China; ^2^ State Key Laboratory of Respiratory Disease Guangzhou China; ^3^ Key Laboratory of Biomaterials of Guangdong Higher Education Institutes, Guangdong Provincial Engineering and Technological Research Center for Drug Carrier Development, Department of Biomedical Engineering Jinan University Guangzhou China

**Keywords:** CDK7, gold nanoparticles, lung adenocarcinoma, M2 macrophages, necroptosis

## Abstract

Although immunotherapy has improved the clinical treatment of lung adenocarcinoma (LUAD), many tumors have poor responses to immunotherapy. In this study, we confirmed that high expression of Cyclin‐Dependent Kinase 7 (CDK7) promoted an immunosuppressive macrophage phenotype and macrophage infiltration in LUAD. Thus, we have developed an internalizing‐RGD (iRGD)‐conjugated gold nanoparticle (AuNP) system which carries siCDK7 to activate the antitumor immune response. The iRGD‐conjugated AuNP/siCDK7 system exhibited good tumor targeting performance and photothermal effects. The AuNP/siCDK7 system with excellent biosafety exerted a significant photothermal antitumor effect by inducing tumor cell necroptosis. Furthermore, the AuNP/siCDK7 system ameliorated the immunosuppressive microenvironment and enhanced the efficacy of anti‐PD‐1 treatment by increasing CD8+ T cell infiltration and decreasing M2 macrophage infiltration. Hence, this iRGD‐conjugated AuNP/siCDK7 system is a potential treatment strategy for lung adenocarcinoma, which exerts its effects by triggering tumor cell necroptosis and immunotherapeutic responses.

## 1 INTRODUCTION

Lung cancer is the leading cause of cancer death in the world according to 2020 Cancer Statistics.^1^ Lung adenocarcinoma (LUAD) comprises over 50% of all lung cancer cases and its frequency is increasing. The five‐year survival rate of advanced LUAD patients has been reported to be only approximately 15%.^2^ Anti‐programmed death 1 (PD‐1) or anti‐programmed death ligand 1 (PD‐L1) antibodies have revolutionized the treatment of advanced non‐small‐cell lung cancer (NSCLC).^3^, ^4^ However, classifying cancers based on T‐cell infiltration showed that many cold tumors exhibit poor response to immunotherapy.^5^ Therefore, pursuing more sensitive immunotherapy approaches for LUAD is important for curing this malignant disease.

CDK7 is a key regulator of cell cycle progression and functions as the catalytic core of the CDK‐activating kinase (CAK) complex.^6^, ^7^ CAK is important for RNA polymerase II‐mediated transcription.^8^ CDK7 inhibition has been reported to reduce abnormal cell proliferation associated with lung cancer.^9^, ^10^ A recent study also found that CDK7 inhibition activates CD8+ T cells by triggering an intrinsic antitumor effect in small‐cell lung cancer,^11^ but the mechanism has not been clarified. With an increased understanding of the role of CDK7 in the progression of various tumors, great efforts have been made to inhibit CDK7 function.^12^ On one hand, to date, several inhibitors targeting CDK7 have recently entered clinical trials.^13^ On the other hand, the downregulation of CDK7 expression de novo may become a promising research direction.

Photothermal therapy (PTT) has become a new noninvasive method in cancer treatment that converts light energy to heat energy.^14^, ^15^ Gold nanoparticles (AuNPs) have been regarded as an important photothermal agent due to their low toxicity, high modifiability, chemical stability, and ease of synthesis and functionalization.^16^, ^17^ It has been shown that the PTT effects of AuNPs can induced ROS production which can activate the tumor cell death program.^18^, ^19^, ^20^ AuNPs also can enhance antitumor ability and provoke antitumor immune responses in vivo when modified with inorganic molecular or bioactive substances.^21^, ^22^, ^23^ Furthermore, as potential delivery vehicles, AuNPs can carry siRNA into the tumor site.^24^ In addition, nanomedicine always exhibits cascade amplification effects for cancer therapy.^25^, ^26^

SiRNA modifications have become a very promising therapeutic strategy for anticancer research.^27^, ^28^ According to the properties of AuNPs, we designed a new AuNP/siCDK7 nanosystem that has potential for gene silencing in combination with PTT. Conjugation with polyethylenimine (PEI) and methoxy polyethylene glycol (mPEG) significantly promoted siRNA cellular uptake and increased gene knockdown efficiency.^29^ A tumor‐homing RGD peptide displays strongly bounding ability to cancer cells which overexpress αvβ3 and αvβ5. Internalizing‐RGD (iRGD) that guided complexes binding to tumor vessels and increasing vascular and tissue permeability was utilized as a bullet, whereas the conventional RGD peptide only delivered the compounds to the blood vessels.^30^, ^31^, ^32^

Necroptosis is one critical mechanisms of immunogenic cell death executed by the receptor interacting protein kinase 1 (RIPK1)‐RIPK3‐mixed lineage kinase domain‐like protein (MLKL) signaling cascade.^33^ Necroptosis is characterized by increased plasma membrane permeability is accompanied by the release of damage‐associated molecular patterns (DAMPs) and cytokines, thereby triggering antitumor immune responses.^33^, ^34^

**SCHEME 1:**
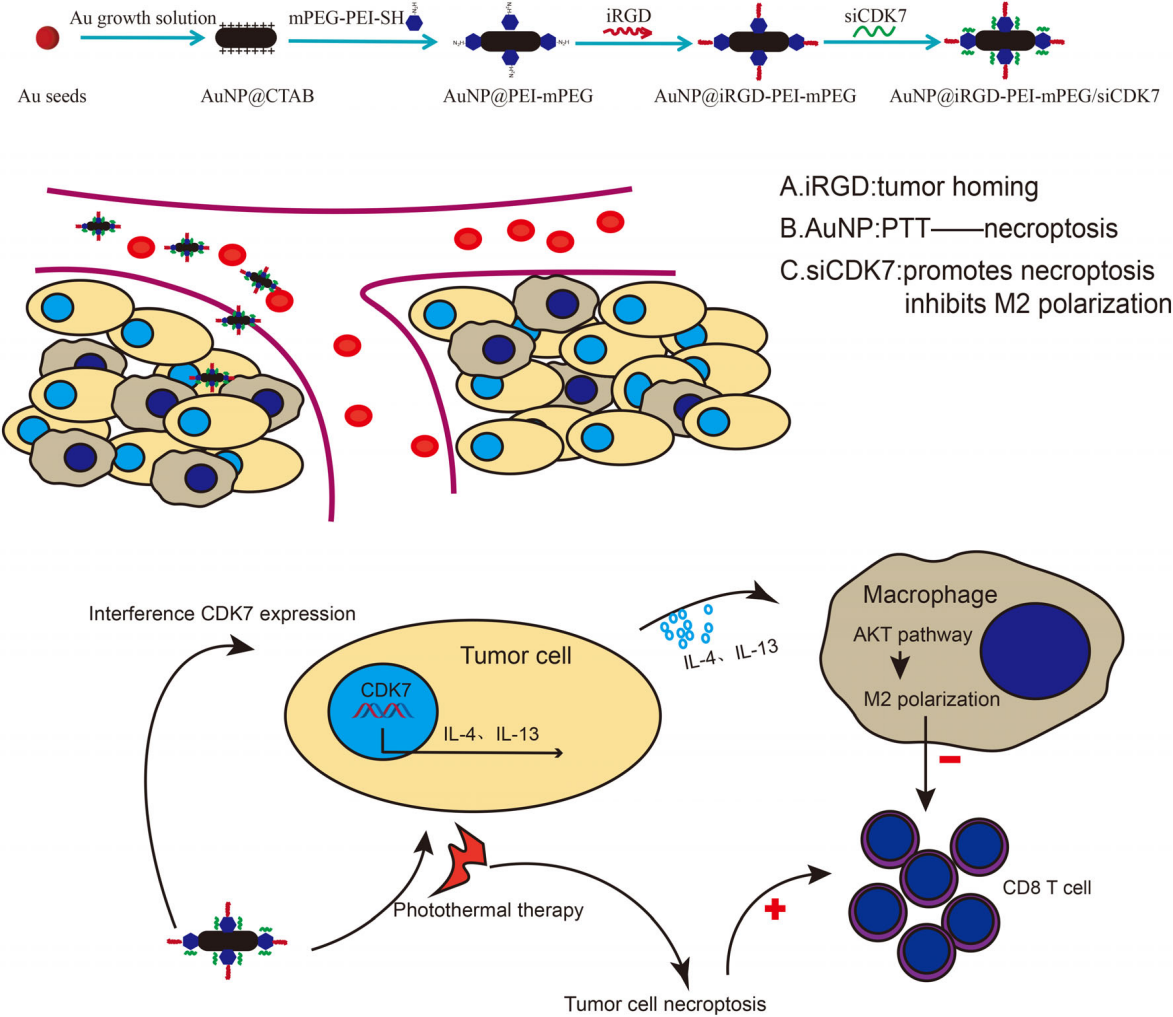
Schematic diagram of nanosystem synthesis steps and antitumor effects

In this research, we studied the effect of CDK7 inhibition in triggering the immune response and developed a new iRGD‐conjugated AuNP@mPEG‐PEI/siCDK7 system to treat LUAD. As shown in Scheme 1, the nanosystem has the following advantages: (1) tumor targeting; (2) inducing tumor cell necroptosis; and (3) inhibiting M2 macrophage polarization and induced immune response.

## 2 RESULTS

### 2.1 CDK7 promoted an immunosuppressive macrophage phenotype

**FIGURE 1:**
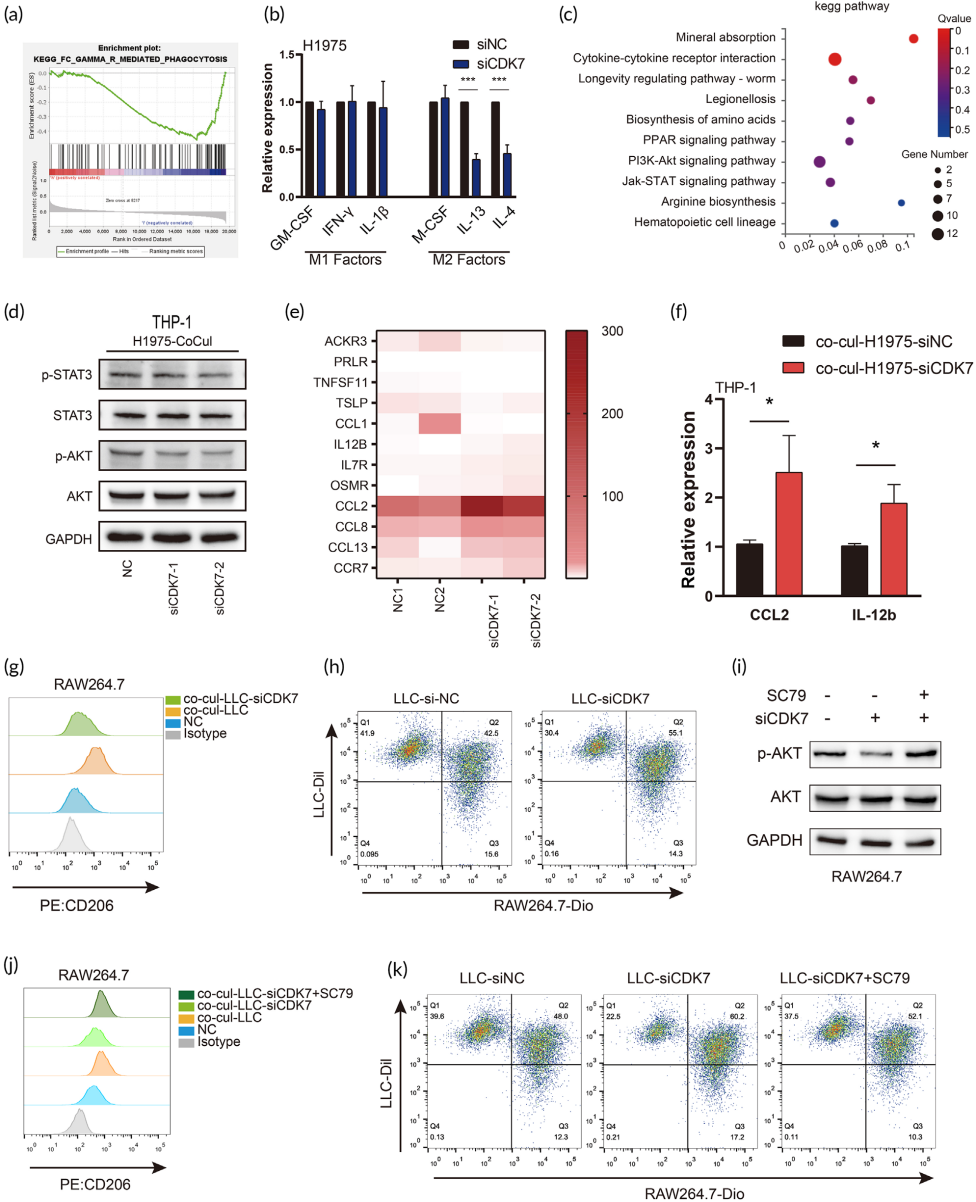
CDK7 promoted the immunosuppressive phenotype of macrophages. (a) GSEA plot of the inhibition of FC gamma R‐mediated phagocytosis in the high CDK7 expression group. (b) The expression of factors associated with M1 and M2 polarization in H1975 cells was measured by qPCR. (c) The top 10 enriched KEGG pathways were considerably associated with different genes in THP‐1 cells cocultured with siNC or siCDK7 H1975 cells. (d) THP‐1 cells were cocultured with H1975 cells. The expression of phosphorylated and total AKT and STAT3 in THP‐1 cells was analyzed by western blotting. (e) Heatmap of differentially expressed genes related to cytokine–cytokine receptor interactions in THP‐1 cells cocultured with siNC or siCDK7 H1975 cells. (f) QPCR showed a trend toward an increase in CCL2 and IL‐12b expression in THP‐1 cells cocultured with CDK7‐knockdown H1975 cells. (g) FCM analysis of CD206 expression in RAW264.7 cells cocultured with or without LLC cells. (h) FCM analysis shows the percentage of phagocytic macrophages in a coculture of RAW264.7 cells with LLC cells. RAW264.7 cells were cocultured with LLC cells and were pretreated with an Akt activator (SC79). (i) The expression of phosphorylated and total AKT was analyzed by western blotting in RAW264.7 cells. (j) FCM analysis of CD206 expression in RAW264.7 cells cocultured with LLC cells. (k) FCM analysis shows the percentage of phagocytic macrophages in a coculture of RAW264.7 cells with LLC cells.

The Cancer Genome Atlas (TCGA) dataset was used to explore the genes whose expression were correlated with CDK7 expression in LUAD tissues. Gene set enrichment analysis (GSEA) was performed. We found that the genes, whose expression levels were negatively correlated with CDK7 expression levels, were mainly enriched in Fc gamma R‐mediated phagocytosis (Figure 1a). Furthermore, we detected the expression level of stimulating factors that promoted M1 and M2 macrophage polarization in LUAD cells. Interference with CDK7 expression significantly downregulated IL‐4 and IL‐13 expression in LUAD cells (Figures 1b and S1A). Next, we investigated the function of CDK7 in macrophages. We cocultured THP‐1 cells with H1975 cells for 48 h, and the THP‐1 cells were subsequently subjected to RNA‐sequence analysis. To characterize the functions of the differentially expressed genes (DEGs) between the H1975‐siNC and H1975‐siCDK7 groups, KEGG pathway analyses were performed. The functions of the DEGs were mainly involved in cytokine–cytokine receptor interactions and the PI3K/AKT and JAK/STAT pathways (Figure 1c). Western blotting (WB) analysis revealed that AKT phosphorylation, but not STAT phosphorylation, was inhibited in siCDK7‐transfected LUAD cells cocultured with macrophages (Figures 1d and S1B). RNA‐sequence analysis also showed that THP‐1 cocultured with CDK7‐knockdown H1975 cells produced more proinflammatory factors and fewer anti‐inflammatory factors (Figure 1e) compared with those cocultured with NC H1975 cells. Consistently, qPCR showed a trend toward an increase in C‐C Motif Chemokine Ligand 2 (CCL2) and IL‐12b expression in macrophages (Figures 1f and S1C) cocultured with CDK7‐knockdown LUAD cells. Flow cytometry (FCM) analysis also showed that RAW264.7 cells cocultured with CDK7‐knockdown LLC cells exhibited a CD206^low^ phenotype (Figure 1g) compared with those cocultured with NC LLC cells. The RAW264.7 cells were stained with Dio, and the LLC cells were stained with Dil. After 6 h of coculture, we observed that RAW264.7 cells phagocytosed the LLC cells (double‐positive cells) (Figure S1D). RAW264.7 cells cocultured with siCDK7‐transfected LLC cells had a significantly higher phagocytic index than those cocultured with siNC‐transfected LLC cells (Figure 1h). An Akt activator reversed the changes in Akt phosphorylation (Figure 1i), M2 polarization (Figure 1j) and phagocytic ability (Figure 1k) in RAW264.7 cells induced by coculture with siCDK7‐transfected LLC cells. The results suggested that Akt activation may be involved in the CDK7‐induced immunosuppressive macrophage phenotype. Thus, inhibiting CDK7 in cancer cells may be a potential approach to activate the macrophage immune response.

### 2.2 CDK7 expression is associated with M2 macrophage infiltration in LUAD

**FIGURE 2:**
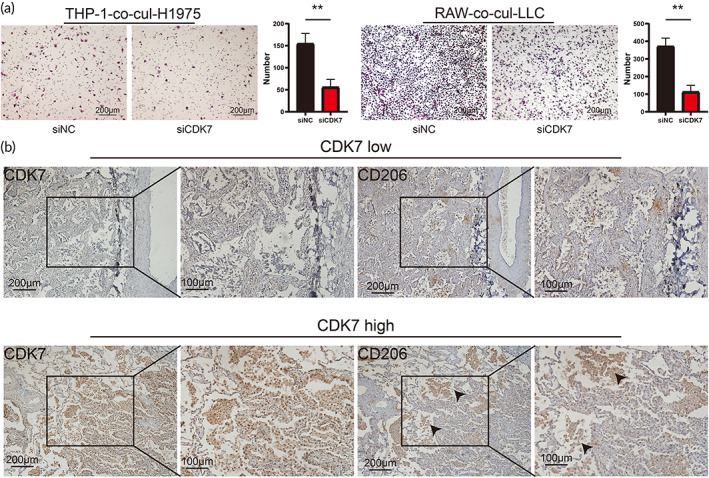
CDK7 expression was associated with M2 macrophage infiltration in LUAD. (a) Representative Transwell images and quantification of the relative migration of macrophages after coculture with tumor cells with different levels of CDK7 expression. (b) The IHC showed a positive correlation between CDK7 expression and M2 macrophage infiltration in LUAD tissues.

To explore whether CDK7 affects the chemotactic migration of macrophages, we conducted a migration assay. CDK7‐knockdown LUAD cells cocultured with macrophages inhibited the chemotactic migration of macrophages toward LUAD cells (Figure 2a). To further confirm this phenomenon, we measured the expression of CDK7 and a biomarker of M2 macrophages (CD206) in 43 LUAD tissues by immunohistochemistry (IHC) (Figure 2b). The results showed a positive correlation between CDK7 expression and M2 macrophage infiltration in LUAD tissues (Table 1).

**TABLE 1:** Correlation between the expression of CDK7 and the abundance of CD206 macrophage

	CDK7 low	CDK7 high	*χ* ^2^	*p* value
CD206 low	12	6	11.5	0.001
CD206 high	4	21		

### 2.3 Synthesis and characterization of the AuNP@mPEG‐PEI‐iRGD nanosystem

**FIGURE 3:**
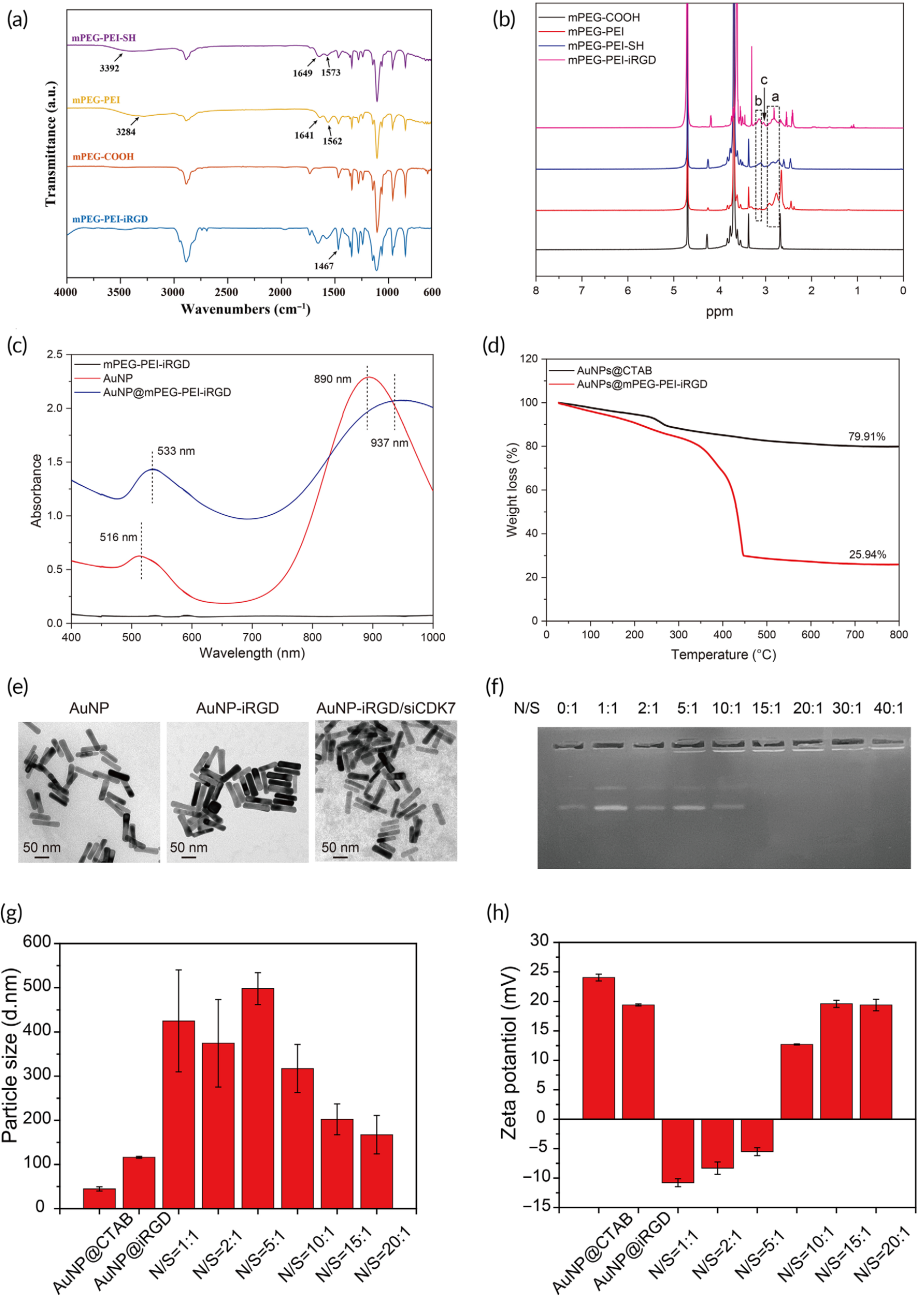
Physicochemical characterization of AuNP@mPEG‐PEI and AuNP@mPEG‐PEI‐iRGD. (a) FTIR spectra of AuNP@mPEG‐PEI and AuNP@mPEG‐PEI‐iRGD. (b) 1H‐NMR of AuNP@mPEG‐PEI and AuNP@mPEG‐PEI‐iRGD. (c) UV–vis spectra of AuNP@mPEG‐PEI and AuNP@mPEG‐PEI‐iRGD. (d) TG curves of AuNP@mPEG‐PEI and AuNP@mPEG‐PEI‐iRGD. (e) TEM images of AuNP, AuNP@mPEG‐PEI‐iRGD and AuNP@mPEG‐PEI‐iRGD/siCDK7. (f) Gel retardation assay of AuNP@mPEG‐PEI‐iRGD/siCDK7 with different N/S ratios. Hydrodynamic diameter (g) and ζ‐potential (h) results of AuNP@mPEG‐PEI‐iRGD/siCDK7 with different N/S ratios

The synthetic route is described in Scheme 1, and the resultant product was validated by ^1^H‐NMR and Fourier transform infrared (FTIR) measurements, as shown in Figure 3a,b. The results indicated the successful synthesis of mPEG‐PEI‐iRGD. We further characterized the obtained AuNP@mPEG‐PEI‐iRGD nanosystem. The UV–vis spectrum of Au@CTAB showed typical absorption bands at approximately 516 and 890 nm, indicating the presence of AuNPs (Figure 3c). After conjugation with mPEG‐PEI‐iRGD, the absorption bands were redshifted to 533 nm and 937 nm (Figure 3c). Thermogravimetric (TG) measurements showed that Au accounted for 79.91%–25.94% of the whole nanosystem when conjugated with mPEG‐PEI‐iRGD (Figure 3d), and the density of iRGD on AuNPs is 8.04%. As revealed by TEM, AuNP@CTAB exhibited characteristics of regular rod‐like morphology and good dispersion (Figure 3e). After modification and conjugation with mPEG‐PEI‐iRGD and siCDK7, AuNPs were in a less well‐dispersed state (Figure 3e).

### 2.4 Preparation and characterization of AuNP@mPEG‐PEI‐iRGD/siCDK7 complexes

We evaluated the condensation ability of AuNP@mPEG‐PEI‐iRGD toward siCDK7 by performing an agarose gel retardation assay. AuNP@mPEG‐PEI‐iRGD/siCDK7 complexes with different N/S ratios were prepared. The results showed that siCDK7 was completely inhibited at an N/S ratio of 20:1 (Figure 3f). Next, we monitored changes in the Dh and ζ‐potential of AuNP@mPEG‐PEI‐iRGD/siCDK7 complexes with N/S values ranging from 1:1 to 20:1. The Dh of AuNP@mPEG‐PEI‐iRGD/siCDK7 complexes gradually decreased as the N/S ratio increased. Dh decreased to 167 nm at an N/S ratio of 20:1 (Figure 3g), indicating that AuNP@mPEG‐PEI‐iRGD/siCDK7 formed a stable complex under this condition. The ζ‐potential of AuNP@mPEG‐PEI‐iRGD/siCDK7 complexes gradually increased when the N/S ratio increased. The ζ‐potential returned to basal levels when the N/S ratio was greater than 15:1 (Figure 3h). We measured the average particle size to test the stability of AuNP/siCDK7 complex in different media. As shown in Figure S2A, the particle size of the complex remained stable in H_2_O and DMEM. The particle size in PBS decreased rapidly in the first day and remained stable in the following days. The protective effect of AuNPs on siCDK7 were also tested by UV absorption. The UV absorption spectra results showed that the absorbance of siCDK7 did not change significantly after laser irradiation (Figure S2B), indicating that the heat produced by AuNPs did not cause much degradation of siCDK7. In addition， as shown in UV spectra, the absorption peak of AuNP@mPEG‐PEI‐iRGD/siCDK7 group in RNase A solution was weakened after 24 h, but still maintained a high absorption peak (Figure S2C). The above results suggested that AuNP@mPEG‐PEI‐iRGD combined with siCDK7 formed positively charged and stable complexes, which facilitated the complexes of entry into cells.

### 2.5 Photothermal properties and CT imaging of AuNP@mPEG‐PEI‐iRGD

**FIGURE 4:**
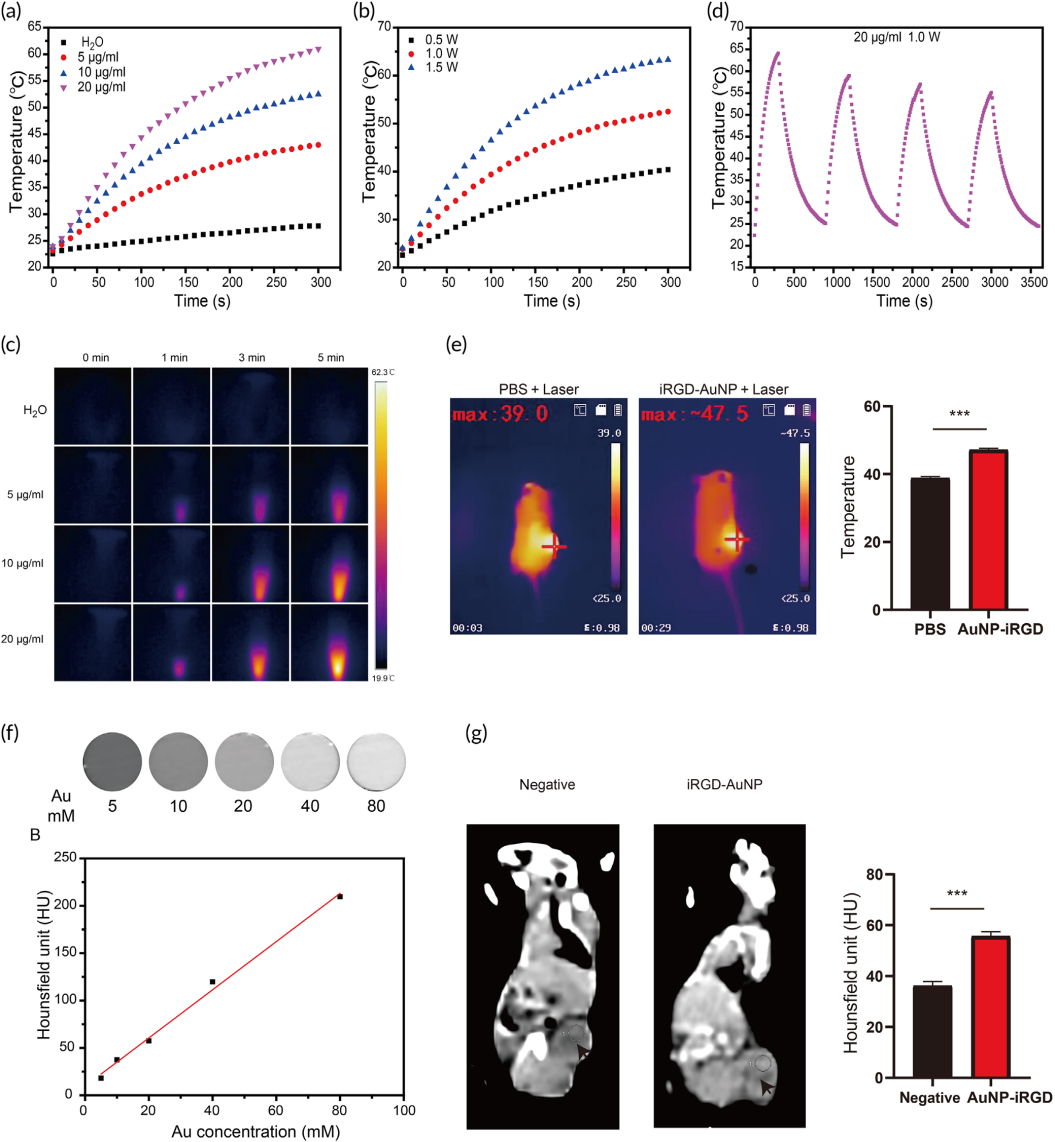
CT imaging and photothermal properties of AuNP@mPEG‐PEI‐iRGD. (a) Temperature change in groups treated with different AuNP@mPEG‐PEI‐iRGD concentrations after 808 nm laser irradiation for different time intervals. (b) Temperature change in groups treated with AuNP@mPEG‐PEI‐iRGD upon 808 nm laser irradiation at different powers for different time intervals. (c) Thermal imaging of groups treated with AuNP@mPEG‐PEI‐iRGD at different concentrations or times. (d) Photothermal stability of AuNP@mPEG‐PEI‐iRGD after four cycles of 808 nm laser irradiation and cooling at room temperature. (e) Thermal imaging of mice after intravenous injection of AuNP@mPEG‐PEI‐iRGD complexes. (f) CT images of groups treated with AuNP@mPEG‐PEI‐iRGD at different concentrations. (g) CT images of mice after intravenous injection of AuNP@mPEG‐PEI‐iRGD complexes

A photothermal imaging system was used to record the temperature changes that occurred with increasing Au concentration or power density during laser irradiation. The results indicated that AuNP@mPEG‐PEI‐iRGD has concentration‐dependent and laser‐power‐density‐dependent photothermal characteristics (Figure 4a–c). Moreover, after four cycles of laser heating and cooling, the photothermal properties of AuNP@mPEG‐PEI‐iRGD were not notably attenuated, which confirmed the photothermal stability of the nanocomplexes (Figure 4d). We also investigated the PTT of AuNP@mPEG‐PEI‐iRGD complexes by performing irradiation with an 808 nm laser in vivo. After irradiation for 5 min, the tumor area in the control and AuNP@mPEG‐PEI‐iRGD groups had temperatures of 39.0 and 47.5°C, respectively (Figure 4e), which were sufficient to achieve PTT effects in the AuNP@mPEG‐PEI‐iRGD group. Due to the high atomic number, AuNPs have been considered as potential CT imaging contrast agents. Therefore, we measured the x‐ray attenuation intensity of AuNP@mPEG‐PEI‐iRGD. CT images became gradually brighter with increasing Au concentrations. We also measured the x‐ray attenuation intensity of AuNP@mPEG‐PEI‐iRGD with different Au concentrations (Figure 4f). A linear relationship was observed between the HU value and Au concentration. The potential of using this system as a contrast agent for CT imaging in a xenografted tumor model was evaluated. CT plain scan axial images were recorded 24 h after injection. The brightness clearly showed great increase in the tumor sites of the AuNP group (Figure 4g).

### 2.6 Gene transfection efficiency in vitro

**FIGURE 5:**
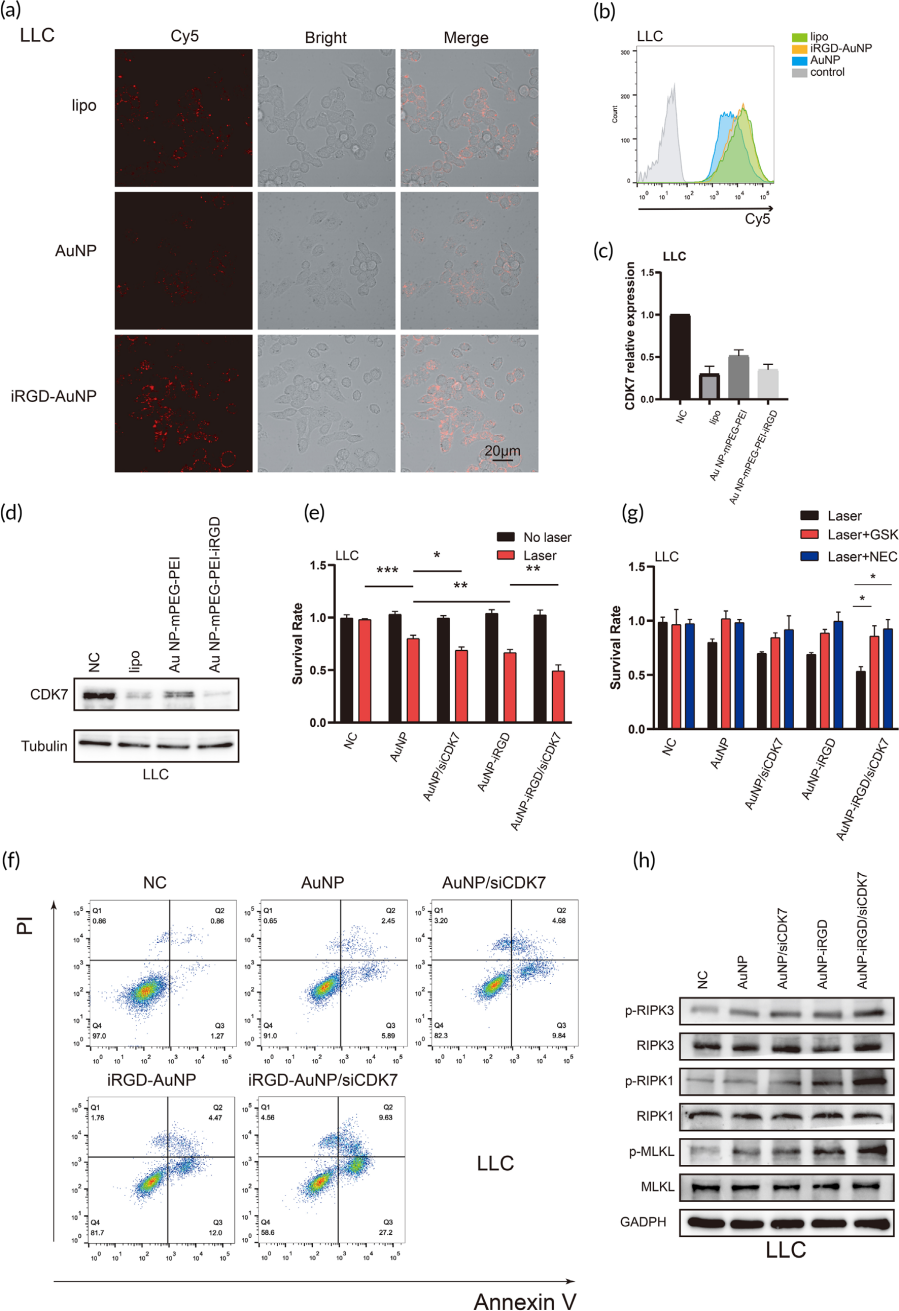
Detection of antitumor effects in LLC cells in vitro. (a) Confocal images of LLC cells treated with lipo/siCDK7, AuNP@mPEG‐PEI/siCDK7 or AuNP@mPEG‐PEI‐iRGD/siCDK7. SiCDK7 was labeled with Cy5. (b) FCM analysis result of LLC cells after treatment with free siCDK7, lipo/siCDK7, AuNP@mPEG‐PEI/siCDK7 or AuNP@mPEG‐PEI‐iRGD/siCDK7 for 48 h. SiCDK7 was labeled with Cy5. Relative mRNA expression (c) and protein expression (d) of CDK7 in LLC cells after treatment with lipo/siNC, lipo/siCDK7, AuNP@mPEG‐PEI/siCDK7 or AuNP@mPEG‐PEI‐iRGD/siCDK7. (e) CCK‐8 assay results showing the relative viability of LLC cells after exposure to different treatments for 24 h with or without laser irradiation. (f) FCM results showing the apoptosis of LLC cells after exposure to different treatments for 24 h with laser irradiation. (g) CCK‐8 assay results showing the relative viability of LLC cells after exposure to different treatments for 24 h with laser irradiation and necroptosis inhibitor treatment. (h) WB results of the expression of necroptosis markers in LLC cells after exposure to different treatments for 24 h with laser irradiation

To confirm the uptake of AuNP@mPEG‐PEI‐iRGD/siCDK7 complexes into tumor cells, siCDK7 was prelabeled with Cy5. The FCM and confocal microscopy results showed that either the Cy5‐labeled Lipo‐siRNA or AuNP@mPEG‐PEI‐iRGD/siRNA group exhibited significantly stronger fluorescence signals than the AuNP@mPEG‐PEI/siCDK7 group (Figures 5a,b and S3A). After proving the successful improvements in the cellular uptake of siCDK7 in the AuNP@mPEG‐PEI‐iRGD‐modified form, the efficiency of CDK7 knockdown was evaluated by quantitative real‐time polymerase chain reaction (qRT–PCR) and WB analysis. The mRNA and protein expression levels of CDK7 showed higher gene transfection efficiency in the Lipo‐siCDK7 and AuNP@mPEG‐PEI‐iRGD/siCDK7 groups than in the AuNP@mPEG‐PEI‐siCDK7 group (Figures 5c,d and S3B, 3C). The above results demonstrated that AuNP@mPEG‐PEI‐iRGD exhibited satisfactory siRNA delivery and interference efficiency.

### 2.7 AuNP complexes induced tumor cell necroptosis via the photothermal effect

The PTT effect of the AuNP/siRNA complex was analyzed by CCK‐8 assay. The results indicated that the AuNP@mPEG‐PEI‐iRGD group was more strongly inhibited than the AuNP@mPEG‐PEI group after laser irradiation (Figures 5e and S3D). We also found that interfering with CDK7 expression successfully enhanced the cell inhibition rate in the AuNP@mPEG‐PEI‐iRGD complex group after laser irradiation (Figures 5e and S3D). To further verify the antitumor efficiency after laser irradiation, the apoptosis and necrosis effects of the AuNP@mPEG‐PEI‐iRGD/siCDK7 complexes were analyzed by FCM assay. The necrosis rate was significantly enhanced in the AuNP@mPEG‐PEI‐iRGD/siCDK7 group compared with the AuNP@mPEG‐PEI‐iRGD or AuNP@mPEG‐PEI/siCDK7 group after laser irradiation (Figures 5f and S3E). The results suggested that interfering with CDK7 expression successfully enhanced the antitumor effects of AuNP‐mPEG‐PEI‐iRGD‐induced PTT.

In addition, we explored the cell death program induced by AuNP@mPEG‐PEI‐iRGD/siCDK7 using restoration and WB assays. After treatment with laser irradiation, necroptosis inhibitors (necrostatin‐1, NEC, 30 μM; GSK‐872, GSK, 3 μM) reduced the cell death induced by AuNP@mPEG‐PEI‐iRGD/siCDK7 after laser irradiation (Figures 5g and S3F). We also found that the levels of phosphorylated necroptosis markers, including receptor interacting protein kinase 1 (RIPK1), RIPK3 and mixed lineage kinase domain‐like pseudokinase (MLKL), were significantly increased in the AuNP@mPEG‐PEI‐iRGD/siCDK7 group (Figures 5h and S3G). Furthermore, we found that necroptosis inhibitors (Nec, 30 μM; GSK, 3 μM) reducing more cell death induced by AuNP@mPEG‐PEI‐iRGD/siCDK7 after laser irradiation compared with apoptosis inhibitors (z‐IETD‐fmk, 20 μM) (Figure S3H, I). In summary, these data demonstrated that AuNP@mPEG‐PEI‐iRGD/siCDK7 complexes induced tumor cell necroptosis after laser irradiation.

### 2.8 In vivo tumor‐targeted photothermal‐gene therapy for LUAD

**FIGURE 6:**
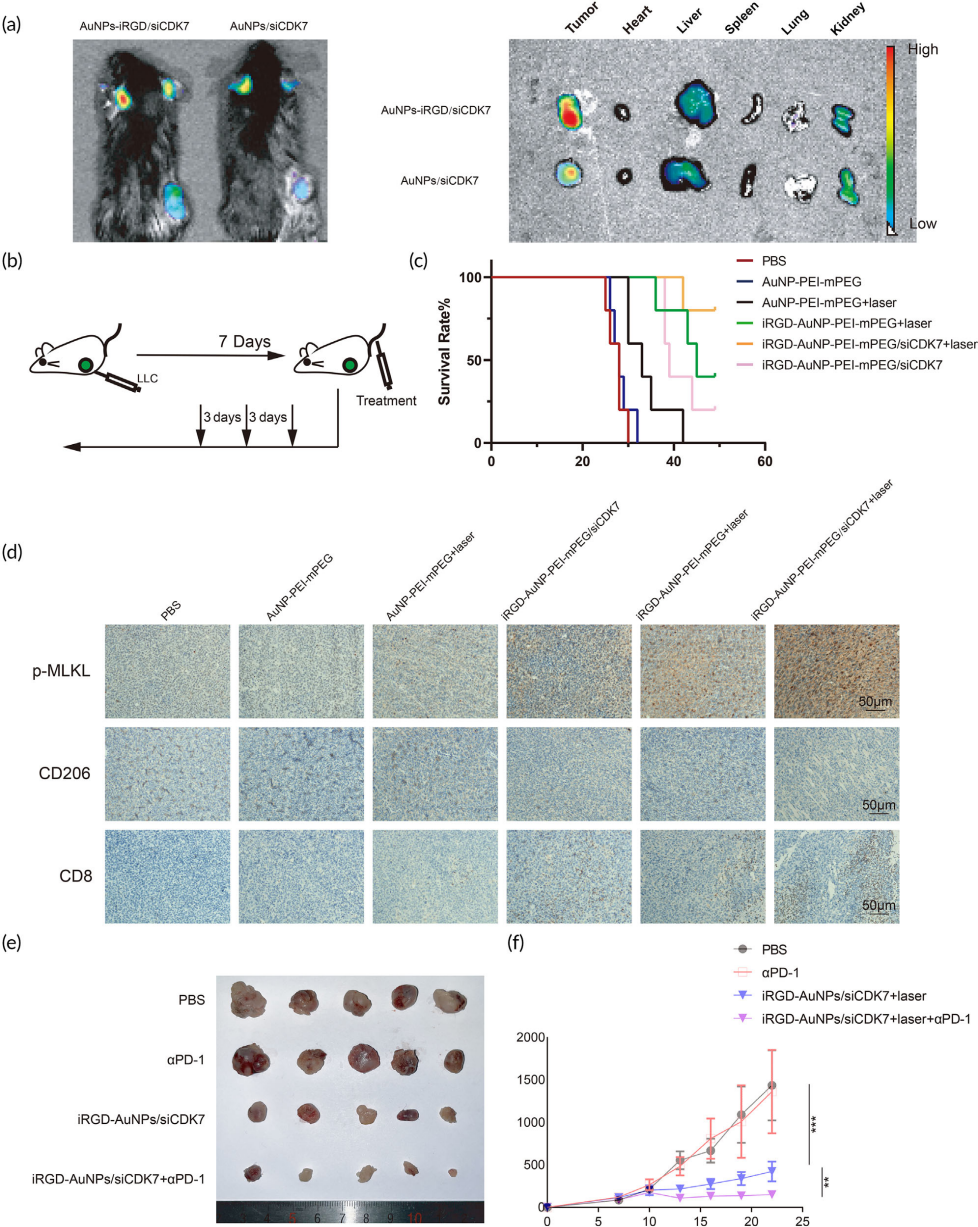
Detection of antitumor effects in vivo. (a) In vivo fluorescent imaging of LLC tumor‐bearing nude mice and their main organs 24 h after intravenous injection of AuNP@mPEG‐PEI/siCDK7 complexes with and without iRGD targeting. Note that siCDK7 was fluorescently labeled with Cy5. (b) Model establishment and treatment schedule of LLC tumor‐bearing mice. (c) Survival curves of LLC tumor‐bearing mice. (d) Immunohistochemical staining of p‐MLKL, CD206 and CD8 in tumor sections from each group after exposure to different treatments for 14 days. (e) Images of tumors isolated from LLC tumor‐bearing mice. (f) Growth curves of tumors from LLC tumor‐bearing mice. αPD‐1 treatment combined with AuNP@mPEG‐PEI‐iRGD/siCDK7 complex treatment exhibited the best antitumor effects.

To assess the tumor‐targeted delivery of siRNA by AuNP@mPEG‐PEI‐iRGD/siCDK7 complexes, we injected Cy5‐labeled AuNP@mPEG‐PEI‐iRGD/siCDK7 complexes into LLC tumor‐bearing mice and observed complex distribution. The results showed that fluorescence was observed in the liver and kidney (Figure 6a). This result suggested that the complexes carried by both targeted and nontargeted nanoparticles were distributed in blood‐rich organs. However, when delivery to tumor tissues was assessed, AuNP@mPEG‐PEI‐iRGD/siCDK7 accumulated to significantly greater degrees in tumors than nontargeted AuNP@mPEG‐PEI/siCDK7 (Figure 6a). To evaluate the antineoplastic therapeutic efficacy of AuNP/siCDK7 complexes, we injected mice with different treatments every 3 days for a total of three injections (Figure 6b). We recorded the overall survival of mice with subcutaneous LLC tumors administered different treatments. The mice treated with PBS or AuNP@mPEG‐PEI without irradiation had shorter survival time (Figure 6c). The AuNP@mPEG‐PEI‐iRGD/siCDK7 group with laser irradiation had prolonged overall survival compared to the other groups (Figure 6c). These data verified that AuNP@mPEG‐PEI‐iRGD/siCDK7 complexes had the best antitumor effect in vivo.

In addition, we tested whether the AuNP/siCDK7 complexes induced tumor cell necroptosis in vivo. As shown in Figure 6d, the level of phosphorylation of the necroptosis marker MLKL in the PBS or AuNP@mPEG‐PEI without irradiation group was low. In contrast, the AuNP@mPEG‐PEI‐iRGD/siCDK7 group with laser irradiation exhibited the highest level of phosphorylated MLKL. To further assess the immune response, we analyzed the numbers of infiltrating CD8+ T cells and CD206 macrophages in the tumor tissues by IHC (Figure 6d). The numbers of CD8+ T cells were markedly increased, while the numbers of CD206 macrophages were decreased after combination treatment with AuNP@mPEG‐PEI‐iRGD/siCDK7 and laser irradiation (Figure 6d). These results indicated that AuNP/siRNA complexes promoted tumor cell necroptosis and enhanced the antitumor immune response by increasing CD8+ T‐cell infiltration and inhibiting CD206 macrophage infiltration into tumors.

We then conducted another subcutaneous tumor assay to study whether AuNP@mPEG‐PEI‐iRGD/siCDK7 complexes enhanced sensitivity to αPD‐1 treatment in vivo. Seven days after the subcutaneous injection of tumor cells (tumor volume was approximately 100 mm^3^), the mice were treated with AuNP@mPEG‐PEI‐iRGD/siCDK7 and/or αPD‐1. In the subcutaneous tumor model, αPD‐1 monotherapy did not inhibit tumor growth or reduce tumor burden, but αPD‐1 treatment combined with AuNP@mPEG‐PEI‐iRGD/siCDK7 complex treatment and laser irradiation effectively inhibited tumor growth (Figure 6e,f). The results further demonstrated that AuNP@mPEG‐PEI‐iRGD/siCDK7 complexes with laser irradiation enhanced the antitumor immune response.

### 2.9 Biological safety evaluation in vivo

Biological safety is a prerequisite for the application of materials in nanomedicine. Therefore, the toxic effects of AuNP/siCDK7 complexes on mouse main organs and blood in vivo were investigated. HE images of the main organs of mice after 14 days of different treatments are shown in Figure S4A; no obvious damage to the tissue morphology and structure of the organs were observed in any of the treatment groups. Blood chemistry parameters for evaluating liver function, the aspartate aminotransferase (AST) and alanine aminotransferase (ALT) levels, and kidney function, the blood urea nitrogen (BUN) levels, in each group were also tested (Figure S4B). Compared with healthy mice, PBS‐treated controls and mice treated with AuNP/siCDK7 showed no significant changes in blood parameters. Therefore, we concluded that AuNP/siCDK7 complexes are safe materials and promising for use in vivo.

## 3 DISCUSSION

Although immunotherapy has been considered as first‐line treatment for NSCLC, a significant portion of patients still show no response to immunotherapy.^35^ To solve this problem, immunotherapy combined with other treatments has been widely explored, thus increasing the number of patients who respond to immunotherapy.^36^ Tumor‐associated macrophages (TAMs) can account for up to 50% of some solid neoplasms, and M2 macrophages often play an immunosuppressive role in the tumor microenvironment (TME).^37^, ^38^ Thus, TAMs could be a promising therapeutic target in the future.

CDK7 play a critical role in mediating the transcription of key cancer dependence genes.^39^ It has been demonstrated that CDK7 inhibition disrupted cell‐cycle progression and induces DNA replication stress and genome instability in lung cancer while triggering immune‐response signaling.^11^ In addition, it has been reported that the high expression of CDK7 is positively correlated with poor survival rate in lung cancer.^40^ In our study we first studied the association between CDK7 expression and the macrophage immune response in LUAD. Interfering with CDK7 expression in LUAD cells decreased the expression of M2 macrophage‐stimulating factors, such as IL‐4 and IL‐13.^41^ This might explain why CDK7 promotes M2 macrophage polarization and infiltration and inhibits macrophage phagocytic abilities in LUAD. Phosphorylation of AKT and STAT are the most well‐known pathways associated with macrophage M2 polarization.^42^, ^43^ We found that interfering with CDK7 expression in LUAD cells cocultured with macrophages inhibited the AKT pathway, but not the STAT pathway, in macrophages. Moreover, it has been reported that the inhibition of macrophage M2 polarization enhances the antitumor immune response and consequently induces greater proinflammatory factor secretion.^44^, ^45^ Consistently, we showed that CDK7 inhibited the expression of proinflammatory factors, such as IL‐12b and CCL2, by macrophages.^46^, ^47^, ^48^ Our work first suggested that downregulation of CDK7 expression enhanced the immune response of macrophages.

To inhibit CDK7 expression in tumors, we synthesized a tumor‐targeted AuNPs system to deliver siRNA. AuNPs exert multifunctional therapeutic effects in cancer treatment via photothermal effects and controllable delivery of their cargo.^49^, ^50^ PEI is a recognized standard contrast polymer for gene delivery. When the PEI/siRNA complex is endocytosed by cells, it will form connotation bodies. Then siRNA will be released into the cytoplasm to play a transfection role.^51^ After the modification of PEI with mPEG, the transfection efficiency and safety of PEI will be enhanced.^29^ Recently, AuNP‐delivered siRNA was applied to treat glioblastoma in a first‐in‐human phase 0 clinical study, further indicating the clinical translational potential of AuNP‐based siRNA delivery systems.^52^ We demonstrated that the AuNP/siCDK7 system could be taken up by tumor cells and had satisfactory RNA interference efficiency and photothermal effects. The findings also demonstrated that the iRGD peptide had the ability to enhance the efficiency of cargo delivery by nanoparticles in vitro and in vivo, which is consistent with previous findings in other tumor models.^53^, ^54^, ^55^ Meanwhile, noninvasive or invasive diagnosis are both key methods to assess the efficacy of treatment.^56^, ^57^ Our synthesized AuNPs had CT imaging capability in the tumor area which could guiding tumor therapy.

As an effective nanodelivery system, the nanosystem in this study could overcome the multiple obstacles to tumor treatments.^58^, ^59^ AuNPs/siCDK7 complexes were able to “kill two birds with one stone”; they directly killed tumor cells and enhanced antitumor immune responses. First, after the AuNPs were loaded with siCDK7, the complexes exhibited good antitumor efficacy and induced tumor cell necroptosis. As one of the critical mechanisms underlying immunogenic cell death, necroptosis has been shown to be involved in the activation of the immune system, particularly antigen presentation and T‐cell responses.^60^, ^61^ Second, regarding changes in the immune landscape, IHC of tumor tissues revealed a substantial decrease in the infiltration of M2 macrophages and an increase in the infiltration of CD8+ T cells after AuNP/siCDK7 complex treatment. These changes in the TME can be explained by tumor cell necroptosis and the inhibition of CDK7 expression, which induced M2 macrophage depolarization. M2 macrophage polarization plays a negative regulatory role in cytotoxic T‐cell recruitment and activation in tumors.^62^, ^63^, ^64^

## 4 CONCLUSIONS

Our findings suggest that CDK7 promotes an immunosuppressive macrophage phenotype in LUAD. CDK7 drove LUAD cells to promote M2 macrophage polarization and inhibited proinflammatory factors secretion by macrophages. Furthermore, the iRGD‐modified AuNP/siCDK7 system exhibited good targeting properties, strongly induced tumor cell necroptosis and greatly improved the immunosuppressive microenvironment. The mechanism of activation T cell immune response by AuNPs/siCDK7 system need to further explore. In general, our data showed the feasibility of targeting multiple pathways to reactivate the antitumor immune response with the tumor‐targeting AuNP/siCDK7 system, and our data provide a new perspective for tumor immunotherapy.

## 5 MATERIALS AND METHODS

### 5.1 Tissue samples

A total of 43 patients diagnosed with LUAD at the Affiliated Cancer Hospital & Institute of Guangzhou Medical University (Guangzhou City, Guangdong Province, China) from 2020 to 2021 were enrolled in this study. None of the patients had received radiotherapy or chemotherapy before surgery. LUAD tumor tissues were collected. All the patients provided written informed consent. The use of human tissues was approved by the Medical Ethical Committee of the Affiliated Cancer Hospital & Institute of Guangzhou Medical University. Pathological TNM staging was assessed according to the American Joint Committee on Cancer (AJCC).

### 5.2 Cell culture

The LLC, H1975 and monocyte THP‐1 and RAW264.7 cell lines were grown in 10% fetal bovine serum (FBS) and 1% penicillin and streptomycin. The cell lines were maintained in an atmosphere of 5% CO_2_ at 37°C.

### 5.3 Immunohistochemistry

The protein expression in tumor tissues was measured using an immunoperoxidase method. Slides were incubated with primary antibodies, followed by incubation with secondary antibodies and being stained with a DAB kit. The IHC intensity of each tissue slide was independently evaluated by two experienced clinical pathologists who were blinded to the patients' clinical data.

### 5.4 Synthesis of AuNPs

Gold nano seeds: A volume of 7.5 ml cetyl trimethyl ammonium bromide (CTAB) (0.2 M) was mixed with 2.5 ml of HAuCl_4_ (0.001 M), and 0.6 ml of frozen sodium borohydride (NaBH_4_) (0.01 M) was added dropwise.

AuNP@CTAB preparation: Fifty milliliters of CTAB (0.2 M) was mixed with 50 ml of HAuCl4 (0.001 M). Then, 1 ml of AgNO_3_ (0.004 M) was added to the mixture, followed by 700 μl of ascorbic acid (AA) (0.0788 M) and 800 μl of HCL (1 M). Finally, the mixture was treated with 80 μl of gold nano seeds and incubated overnight.

Synthesis of mPEG‐PEI. One hundred milligrams of mPEG‐COOH was first dissolved in 10 ml of deionized water, followed by the addition of 10 mg of 1‐ethyl‐3‐(3‐dimethylaminopropyl) carbodiimide hydrochloride (EDC). Then, the pH was adjusted to 7.0. Then, 15 mg of N‐hydroxysuccinimide (NHS) and 100 mg of PEI were added. After 8 h of incubation, the solution described above was dialyzed against water using a dialysis membrane (MW 3500 Da) for 1 day and finally lyophilized to obtain mPEG‐PEI.

Synthesis of mPEG‐PEI‐SH. Two milligrams of 3‐mercaptopropionic acid was first dissolved in 5 ml of deionized water, followed by the addition of 2 mg of EDC. Then, the pH was adjusted to 7.0. This step was followed by the addition of 3 mg NHS and 40 mg mPEG‐PEI. After 8 h of incubation, the solution described above was dialyzed against water using a dialysis membrane (MW 3500 Da) for 1 day and finally lyophilized to obtain mPEG‐PEI‐SH.

Synthesis of mPEG‐PEI‐iRGD. Ten milligrams of iRGD‐COOH was first dissolved in 10 ml of deionized water, followed by the addition of 10 mg of EDC. Then, the pH was adjusted to 7.0. This step was followed by the addition of 15 mg of NHS and 40 mg of mPEG‐PEI‐SH. After 8 h of activation, the above solution was dialyzed against water using a dialysis membrane (MW 3500 Da) for 1 day and finally lyophilized to obtain mPEG‐PEI‐iRGD.

Synthesis of AuNP@mPEG‐PEI‐iRGD. Seventy‐five microliters of mPEG‐PEI‐iRGD (0.18 mM) was added to the AuNP solution. The mixture was subjected to ultrasound for 90 min at 40°C. The synthesized AuNP@PEG‐PEI‐iRGD solution was centrifuged, resuspended twice in ultrapure water and stored at 4°C.

Preparation of the AuNPs/siRNA complexes. AuNP‐mPEG‐PEI‐iRGD/siCDK7 complexes were prepared by adding an appropriate amount of siCDK7 to AuNP‐mPEG‐PEI‐iRGD/siCDK7 dispersion and gentle mixing for 30 min. The weight ratio of AuNP‐mPEG‐PEI‐iRGD/siCDK7 was abbreviated as N/S.

### 5.5 Characterization

The absorbance of mPEG‐PEI‐iRGD and AuNPs was determined by UV spectrophotometry in the range of 200–1000 nm. Fourier transform infrared (FTIR) spectra were recorded by an FTIR spectrophotometer in the range of 4000–399 cm^−1^. The morphology of AuNPs was characterized by transmission electron microscopy (TEM). The chemical structures were characterized by 1H NMR spectroscopy using deuterium oxide (D2O) as the solvent and analyzed by MestReNova software. Thermogravimetric (TG) analysis was carried out to determine the compositions of the AuNPs using thermogravimetry. The TG settings were as follows: temperature range of 25–800°C and heating rate of 10°C/min. The ζ‐potential and hydrodynamic diameter were recorded by a Zetasizer Nano ZS (Malvern) apparatus with size and ζ‐potential analyzer software. AuNP@iRGD dispersions with a series of Au concentrations (5 ~ 80 mM) were placed in 0.3 ml Eppendorf tubes, and all the tubes were loaded into the CT imaging system and scanned sequentially. In vivo CT imaging ability was performed using LLC tumor‐bearing mice. The mice were injected with 100 μg/g AuNP@mPEG‐PEI‐iRGD via the tail vein. CT images were captured, and Hounsfield units (HU) were measured.

### 5.6 Agarose gel retardation assay

The siCDK7 complexation ability of AuNP@mPEG‐PEI‐iRGD was evaluated by performing an agarose gel retardation assay. AuNP@mPEG‐PEI‐iRGD/siCDK7 complexes with different N/S ratios were separated by 1% agarose gel at 140 V for 30 min. A fluorescence detector was used to capture images.

### 5.7 Photothermal performance

AuNP@mPEG‐PEI‐iRGD dispersions with a series of Au concentrations were placed in cuvettes. All cuvettes were irradiated with 808 nm near‐infrared light at different powers for 5 min. In vivo PTT was performed using LLC tumor‐bearing mice. The mice were injected with 100 μl of saline or 100 μg/g AuNP@mPEG‐PEI‐iRGD via the tail vein. Twenty‐four hours after injection, the tumor region was irradiated with an 808 nm laser at a power density of 1 w cm^−2^ for 5 min. The temperature was measured by a thermocouple thermometer and captured by a thermal imaging camera.

### 5.8 RNA sequencing analysis

RNA sequencing was performed by Beijing Genomics Institution. Differentially expressed genes (DEGs) were identified with the following parameters: adjusted *p* < 0.05 and log2FC >2. Kyoto Encyclopedia of Genes and Genomes (KEGG) pathway enrichment analysis was used to identify the pathways related to the DEGs.

### 5.9 Quantitative real‐time polymerase chain reaction (qRT–PCR)

Total RNA was isolated from cells by AG RNAex Pro Reagent AG21102 (Accurate Biotechnology [Human] Co., Ltd.). RNA was reversely transcribed into cDNA via Evo M‐MLV RT Premix for qPCR AG11706 (Accurate Biotechnology [Human] Co., Ltd.). The primer sequences are shown in Table S1.

### 5.10 Western blotting analysis

The proteins were separated by 10% SDS–PAGE and transferred to PVDF membranes (Millipore). The membranes were blocked in 5% BSA for 2 h at room temperature. The membranes were washed once for 5 min with PBST and incubated with primary antibodies against Akt (Abcam, ab179463), p‐AKT (Abcam, ab192623), STAT3 (Abcam, ab68153), p‐STAT (Abcam, ab76315), GAPDH (Proteintech, 60004‐1‐Ig), CDK7 (Abcam, ab256787), β‐Tubulin (Proteintech, 10068‐1‐AP), MLKL (Abclonal, A13451), p‐MLKL (Abclonal, AP0949; Boster, P00535), RIPK1 (Abclonal, A7414), p‐RIPK1 (Abclonal, AP1115), RIPK3 (Abclonal, A5431), and p‐RIPK3 (Abcam, ab209384; Abcam, ab222320) overnight at 4°C. The membranes were washed three times for 15 min with PBST and incubated with HRP‐conjugated secondary antibodies for 90 min at room temperature. The membranes were washed three times for 15 min each with PBST and visualized using ECL (Biosharp).

### 5.11 Phagocytosis assay

RAW264.7 cells were stained with DiO, and LLC cells were stained with Dil for 15 min. RAW264.7 cells were incubated with LLC cells exposed to different treatments for 6 h. Finally, the samples were analyzed using flow cytometry (FCM).

### 5.12 Transfection assay

The cellular uptake of AuNP complexes with and without iRGD was analyzed to confirm the targeting ability of iRGD. Cy5‐labeled, lipo‐siCDK7, AuNP@mPEG‐PEI/siCDK7 or AuNP@mPEG‐PEI‐iRGD/siCDK7 complexes were used to treat the cells. The injected amounts of siCDK7 and AuNPs were 0.5 and 10 μg/ml, respectively. After incubation for 4 h, the cells in each group were washed with PBS and cultured in medium supplemented with 10% FBS for 48 h.

### 5.13 Apoptosis assay

The cell apoptosis rate was determined by an Annexin V 633 Apoptosis Detection Kit. The cells were collected and washed twice with cold PBS, followed by incubation with Annexin V/PI for 30 min on ice.

### 5.14 Transwell assay

Transwell chambers were used to investigate cell migration. After digestion and centrifugation, the cells were resuspended in FBS‐free DMEM. A total of 5 × 10^4^ LLC or H1975 cells were added to the lower chamber. A 100 μl RAW264.7 or THP‐1 cell suspension at a density of 3 × 10^4^ cells/ml was placed in the upper chamber. After 24 h, the cells were fixed in 4% polyformaldehyde and stained with 0.1% crystal violet solution. The cells were observed under a light microscope, and five randomly selected fields of view were counted and photographed at a magnification of 200×.

### 5.15 Tumor growth inhibition

For in vivo experiments, a subcutaneous tumor model was established by the subcutaneous injection of LLC tumor cells (1 × 10^6^) into C57BL/6 mice. After 1 week, the mice (*n* = 5) randomly received different treatments every 3 days for a total of three treatments. The injected amounts of materials in terms of siCDK7, AuNP and mAb anti‐mouse PD‐1(αPD‐1, BioXcell, BE0146) were 5, 100, and 10 μg/g, respectively. Survival and tumor growth curves of the tumor‐bearing mice were generated in an independent experiment, and the endpoint of the animal experiment was when the tumor volume reached approximately 2000 mm^3^. The tissues were collected for analysis in further independent experiments after treatment for 2 weeks. All animals were managed according to the Care and Use of Medical Laboratory Animals and all experimental were approved by Institutional Animal Care and Use Committee of Guangzhou Medical University.

### 5.16 Safety evaluation

After treatment for 2 weeks, the main organs (heart, liver, spleen, lung, and kidney) and serum of the mice were collected for further investigation. Major organs were sectioned for HE staining analysis. The serum biochemistry (ALT, AST, BUN) was analyzed using an Automatic Biochemical Analyzer.

## AUTHOR CONTRIBUTIONS

**Rui Cai:** Visualization (lead); writing – original draft (lead). **Meng Wang:** Validation (lead); writing – original draft (supporting). **Meiyuan Liu:** Methodology (equal); validation (equal); writing – original draft (supporting). **Xiongjie Zhu:** Investigation (equal); methodology (equal). **Longbao Feng:** Investigation (equal); methodology (equal); validation (equal). **Zhongjian Yu:** Data curation (equal); resources (equal). **Xia Yang:** Data curation (equal); resources (equal). **Zhiwu Zhang:** Investigation (equal); methodology (equal). **Huili Guo:** Data curation (equal); resources (equal). **Yanfang Zheng:** Funding acquisition (lead); supervision (lead); writing – review and editing (lead).

## FUNDING INFORMATION

This study was supported financially by grants from the Science and Technology Planning Project of Guangdong Province (2020A0505100038, 2021A1515010793, 2020B1111560001), National Natural Science Foundation of China (81974434), the Affiliated Cancer Hospital & Institute of Guangzhou Medical University (2020‐YZ‐01), the Science and Technology Program of Guangzhou City (No. 202201020097), Clinical Key Specialty Construction Project of Guangzhou Medical University (YYPT202017), the Key Areas and Development Program of Guangzhou (202007020006, 202103030003), and the International Science and Technology Cooperation Project of Huangpu District/Guangzhou Development District (2019GH11, 2020GH14).

## CONFLICT OF INTEREST

The authors declare no conflict of interest.

## Supporting information


**Appendix S1**. Supporting Information

## Data Availability

Data available on request from the authors.
