# Supplementary material for: An iRGD‐conjugated photothermal therapy‐responsive gold nanoparticle system carrying siCDK7 induces necroptosis and immunotherapeutic responses in lung adenocarcinoma
Source: Bioeng Transl Med. 2022 Oct 27;8(4):e10430. doi: 10.1002/btm2.10430 (PMC10354770; doi:10.1002/btm2.10430)
Supplement: Supplementary file 1 — Appendix S1. Supporting Information [file BTM2-8-e10430-s001.docx]

**Supporting Information.**


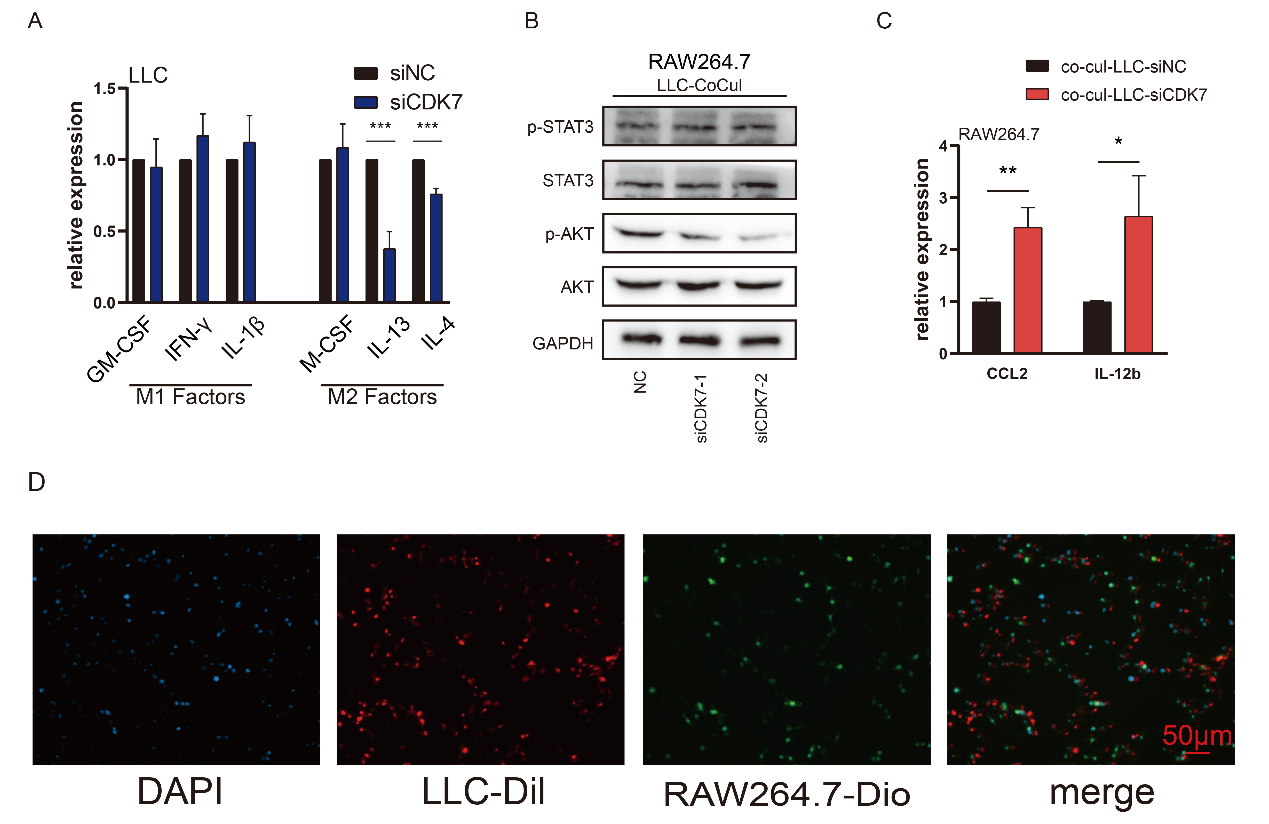
**Figure S1. CDK7 promoted the immunosuppressive phenotype of macrophages.** A. Expression of factors associated with M1 and M2 polarization in LLC cells was measured by qPCR. B. RAW264.7 cells were cocultured with LLC cells. The expression of phosphorylated and total AKT and STAT3 in RAW264.7 cells was analyzed by western blotting. C. QPCR showed a trend toward an increase in CCL2 and IL-12b expression in RAW264.7 cells cocultured with CDK7-knockdown LLC cells. D. Representative fluorescence images for Dil-labelled LLCs and Dio-labelled RAW264.7 cells after 6 h of coculture.


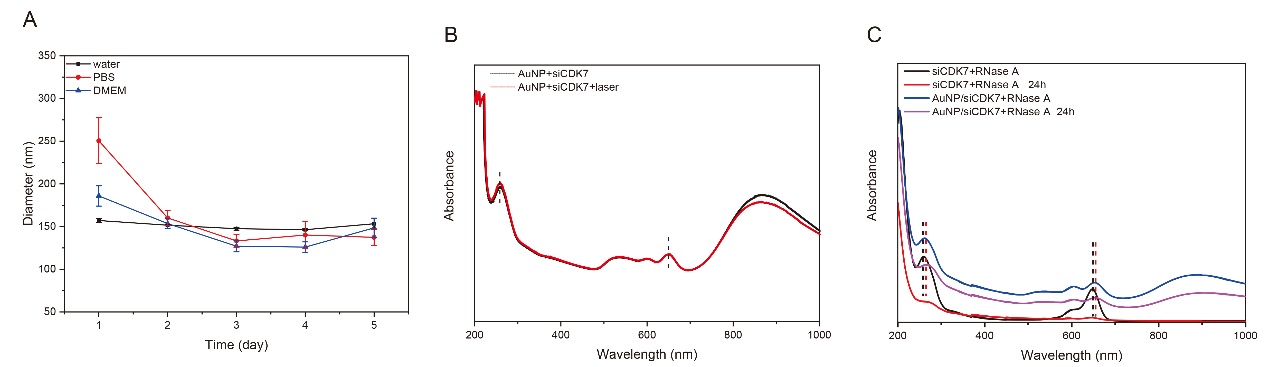


**Figure S2. Stability of AuNP@mPEG-PEI-iRGD/siCDK7.** A. The average particle size of the complex in different media. B. The UV absorption spectra of siCDK7 labeled with fluorescent were tested after irradiated with 808nm near-infrared light. C. The UV absorption spectra of siCDK7 labeled with fluorescent were tested in different solution.


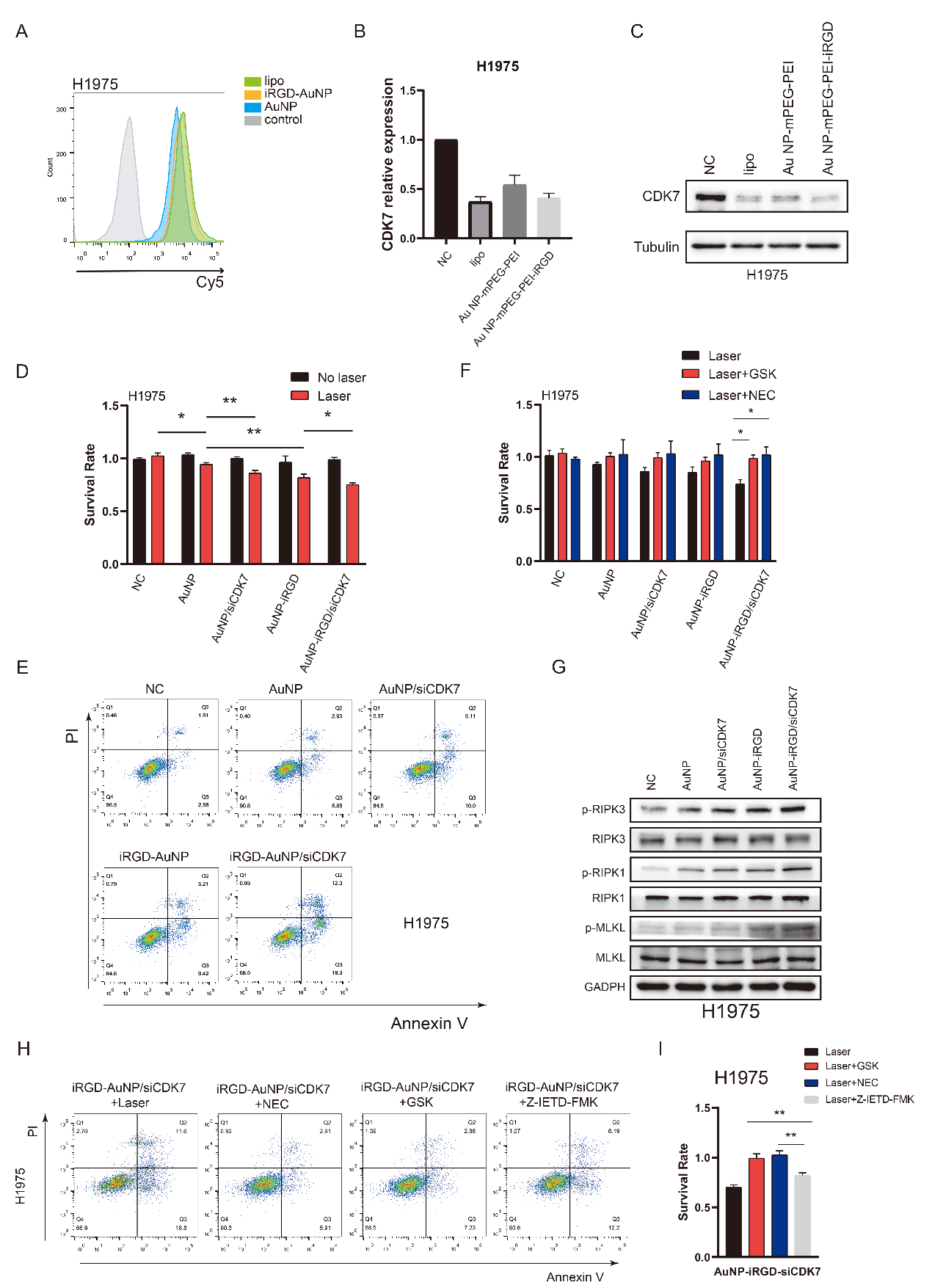


**Figure S3. Detection of antitumor effects in H1975 cells *in vitro*.** A. FCM analysis result of H1975 cells after treatment with free siCDK7, lipo/siCDK7, AuNP@mPEG-PEI/siCDK7 or AuNP@mPEG-PEI-iRGD/siCDK7 for 48 h. SiCDK7 was labeled with Cy5. Relative mRNA expression (B) and protein expression (C) of CDK7 in H1975 cells after treatment with lipo/siNC, lipo/siCDK7, AuNP@mPEG-PEI/siCDK7 or AuNP@mPEG-PEI-iRGD/siCDK7. D. CCK-8 assay results showing the relative viability of H1975 cells after exposure to different treatments for 24 h with or without laser irradiation. E. FCM results showing the apoptosis of H1975 cells after exposure to different treatments for 24 h with laser irradiation. F. CCK-8 assay results showing the relative viability of H1975 cells after exposure to different treatments for 24 h with laser irradiation and necroptosis inhibitor treatment. G. WB results of the expression of necroptosis markers in H1975 cells after exposure to different treatments for 24 h with laser irradiation. FCM analysis (H) and CCK-8 (I) assay results showing the relative viability of H1975 cells after exposure to iRGD-AuNPs/siCDK7 for 24 h with laser irradiation and necroptosis inhibitor or apoptosis inhibitors treatment.


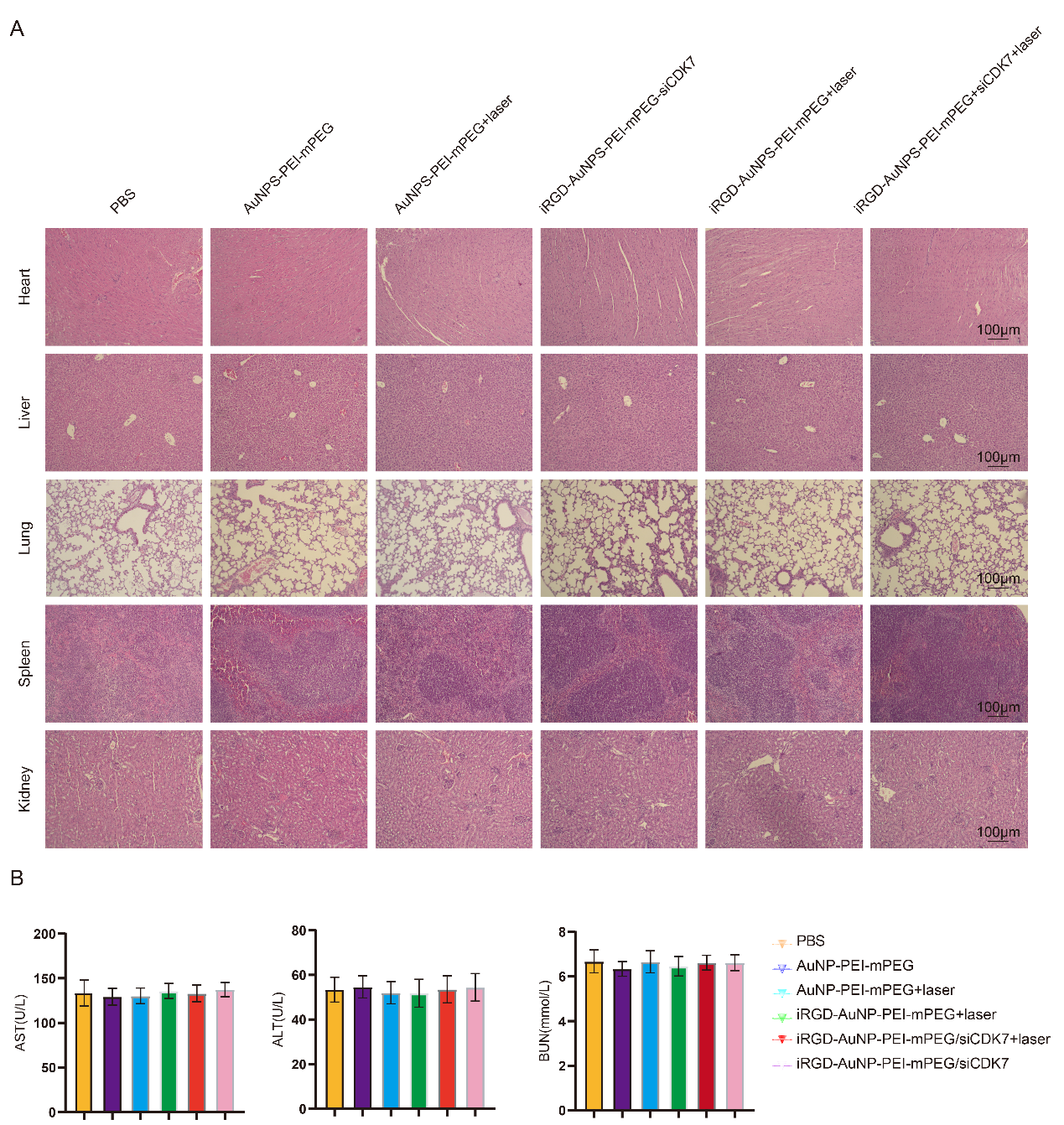


**Figure S4. *In vivo* evaluation of material biosafety.** A. HE staining images of mouse organs after exposure to different treatments for 14 days. B. Plasma parameters were measured to evaluate the functions of the liver and kidney.

**Table S1. The primer** **sequences used for qRT-PCR**

| **Genes** | **Sequence (5’ to 3’)** |
| --- | --- |
| H-CCL2-F | GAAGCTCGCACTCTCGCCTCC |
| H-CCL2-R | TGAGCGAGCCCTTGGGGAATGA |
| M-Ccl2-F | TTAAAAAACCTGGATCGGAACCAA |
| M-Ccl2-R | GCATTAGCTTCAGATTTACGGGT |
| H-IL-12b-F | TGCCCATTGAGGTCATGGTG |
| H-IL-12b-R | CTTGGGTGGGTCAGGTTTGA |
| M-Il-12b-F | GGAGACCCTGCCCATTGAACT |
| M-Il-12b-R | CAACGTTGCATCCTAGGATCG |
| H-GADPH-F | GTGTCGCTGTTGAAGTCAGAG |
| H-GADPH-R | CATCAAGAAGGTGGTGAAGCAG |
| M-Gadph-F | CCACCCCAGCAAGGAGAC |
| M-Gadph-R | GAAATTGTGAGGGAGATGCT |
| H-CDK7-F | GGGCACACCAACTGAGGAACAG |
| H-CDK7-R | CGTCTCCTGCTGCACTGAAGATG |
| M-Cdk7-F | CCAAATCGTCGCCATTAAGAAA |
| M-Cdk7-R | GAGCTTTATCTCCCTTAAGGCT |
